# Impaired myelination in multiple sclerosis organoids: p21 links oligodendrocyte dysfunction to disease subtype

**DOI:** 10.3389/fncel.2026.1786186

**Published:** 2026-03-24

**Authors:** Saud A. Sadiq, Tanmay Mehta, William Holzman, Annie McDermott, Nicolas Daviaud

**Affiliations:** Tisch Multiple Sclerosis Research Center of New York, New York, NY, United States

**Keywords:** E2F1, multiple sclerosis, myelination, oligodendrocytes, organoids, p21, Pak1

## Abstract

Multiple sclerosis (MS) is an autoimmune inflammatory disease of the central nervous system. The cause of the disease is unknown but both genetic and environmental factors are strongly involved in its pathogenesis. We derived cerebral and spinal cord organoids from induced pluripotent stem cells (iPSC) from healthy controls as well as from primary progressive MS (PPMS), secondary progressive MS (SPMS) and relapsing–remitting MS (RRMS) patients to investigate and compare oligodendrocyte differentiation and myelination capacity. In MS organoids, particularly in PPMS, we observed a decrease in p21 expression associated with a dysregulation of PAK1 and E2F1 expression. In parallel, a decrease in oligodendrocyte maturation was detected in long-term cultured cerebral and spinal cord organoids, especially in PPMS, leading to a reduced myelination capacity. Disruption of astrocyte and neuronal populations was also observed. Our findings demonstrate that in MS, inherent deficits in the p21 pathway may alter glial and neuronal cell populations and may contribute to the disease pathogenesis by reducing the capacity for myelin repair.

## Introduction

Multiple sclerosis (MS) is an auto-immune inflammatory disorder that may lead to irreversible neurological disability and cognitive decline (Filippi et al., 2018). It is characterized by widespread focal lesions of primary demyelination in the brain and spinal cord, with variable axonal, neuronal, and astroglial injury. The disease presents primarily as two clinical subtypes: relapsing–remitting MS (RRMS), which accounts for 85–90% of cases which may evolve to secondary progressive MS (SPMS), and primary progressive MS (PPMS) which affects about 10% of cases and is marked by a steady functional decline from disease onset (Lublin and Reingold, 1996; Andersson et al., 1999). The origin, evolution, and physiological basis of MS’s varied phenotypic expressions remain poorly understood in part due to the relative inaccessibility of human brain tissues and limitations of animal models (Ransohoff, 2012). The development of MS is influenced by genetic factors, with familial relatives of patients, especially first-degree relatives, being more susceptible to developing MS compared to the general population. There is also epidemiological evidence that environmental triggers possibly infections are important in disease causation (Gouider et al., 2024). This interplay of genetic susceptibility and environmental factors contributes to autoimmunity and the multifaceted nature of MS (Willer et al., 2003).

We previously described cerebral organoids (c-organoids) as an innovative model to study MS. Using c-organoids derived from induced pluripotent stem cells (iPSCs) of healthy control subjects, and PPMS, SPMS and RRMS patients, we showed a decrease of proliferative capacity, notably in progressive forms of MS. This was associated with a reduction of the progenitor pool and an increase of neurogenesis possibly due to a symmetric shift of the cell division mode. We linked these effects to a strong decrease of p21 expression in PPMS organoids, unrelated to the DNA damage and apoptosis pathway (Daviaud et al., 2023). Interestingly, it has been reported that p21 is required for the differentiation of oligodendrocytes and that animal knockdown for p21 exhibited hypomyelinated brains (Zezula et al., 2001).

Here we further investigated neural and glial cell differentiation and myelination capacity in MS using our cerebral and spinal cord organoid (SCO) models. We detected a significant decrease in oligodendrocyte maturation and myelination capacity in MS organoids, especially in PPMS. Additionally, a defect of astrocyte population and an imbalance of inhibitory/excitatory neurons were found, which are key factors in MS onset and possibly in causing cognitive impairment, physical disability, and fatigue in MS patients. We confirmed that the p21 pathway dysfunction was a critical abnormality in our organoid model of MS, while expression of other Cyclin Dependent Kinase inhibitors (CDKi), such as p16, p27 and p57, was unaltered. Furthermore, we focused on the p21 regulators E2F1 and PAK1. In the oligodendroglial lineage, the transcription factor E2F1 contributes to the transition of oligodendrocyte precursor cells (OPCs) from proliferation to differentiation (Magri et al., 2014), highlighting its role at the interface between cell cycle control and lineage progression. PAK1, a serine/threonine kinase, also participates in oligodendrocyte development. Its activity has been associated with oligodendrocyte differentiation and myelination (Brown et al., 2021), as well as with proper myelin sheath formation in the central nervous system (Thurnherr et al., 2006). Moreover, PAK1 can negatively regulate p21 expression (Wang et al., 2018), thereby promoting cell growth, while p21 itself is transcriptionally regulated by E2F1 (Hiyama et al., 1998). Together, these findings suggest a functional interplay between E2F1, PAK1, and p21 during oligodendrocyte lineage progression but also in regulation of the excitatory/inhibitory network.

In conclusion, this work demonstrates that cortical and SCO derived from patients with MS can be used as an innovative tool to better understand the genetic basis of phenotypic differences seen in MS. This work provides a better understanding of the genetic/epigenetic changes in patients with MS and sheds new light on the developmental aspect of MS, which is of particular interest for understanding the pathogenesis of MS.

## Materials and methods

### Patient selection

Peripheral blood mononuclear cell (PBMCs) samples were collected from healthy control subjects and clinically definite MS patients diagnosed according to the revised 2017 McDonald Criteria. All MS patients underwent neurological examination and MRI imaging and were classified as having RRMS, SPMS or PPMS by board-certified neurologists specializing in MS care. All protocols were IRB approved, and all donors provided written informed consent for participation. Human iPS cells were generated from PBMCs from donor blood samples. Other cell lines were obtained from the New York Stem Cell Foundation. Donor information is summarized in Tables 1, 2.

**Table 1:** Patients’ details.

Sex	Age	Diagnosis	Source
M	28	Control	NYSCF
M	23	Control	Tisch MSRCNY
M	36	Control	Tisch MSRCNY
M	67	Control	Tisch MSRCNY
F	52	Control	Tisch MSRCNY
F	40	Control	Tisch MSRCNY
F	62	PPMS	NYSCF
M	61	PPMS	NYSCF
F	58	PPMS	Tisch MSRCNY
M	40	PPMS	Tisch MSRCNY
F	57	PPMS	Tisch MSRCNY
M	38	RRMS	Tisch MSRCNY
F	67	RRMS	Tisch MSRCNY
M	24	RRMS	NYSCF
F	50	RRMS	NYSCF
M	60	SPMS	Tisch MSRCNY
M	48	SPMS	Tisch MSRCNY
F	54	SPMS	Tisch MSRCNY
F	34	SPMS	Tisch MSRCNY
F	67	SPMS	Tisch MSRCNY

Table summarizing patients’ characteristics such as sex, gender, age, diagnosis, and cell source.

**Table 2:** Patients distribution.

Statistical significance	Healthy controls	PPMS	RRMS	SPMS	
Number of patients	6	5	4	5	
Age at collection, mean (SD), years	41 (16)	55.6 (8.9)	44.7 (18)	53 (13)	n.s.
Gender distribution	2 F; 4 M	3 F; 2 M	2 F; 2 M	3 F; 2 M	n.s.

Table summarizing the age and gender distribution in our cohort. No significant difference was observed in age (One-way ANOVA, *p* = 0.3450) or gender distribution among the different conditions (Chi-square, *p* = 0.7851).

### Reprogramming CD34^+^ progenitor cells

Human PBMCs were isolated according to the “STEMCELL Integrated Workflow for the Isolation, Expansion, and Reprogramming of CD34^+^ Progenitor Cells”. Briefly, blood samples were collected in heparin-vacutainer tubes from donors in amounts ranging from 8 to 20 mL. CD34^+^ hematopoietic stem and progenitor cells were isolated from peripheral blood using the EasySep RosetteSep Kit (STEMCELL) and expanded *in vitro* in CD34^+^ expansion media consisting of StemSpan SFEM II and CD34^+^ expansion supplements (STEMCELL). After 7–10 days of culture, 1×10^6^ cells were collected for reprogramming by electroporation using the Epi5 Episomal iPSC Reprogramming Kit (ThermoFisher) and Human CD34^+^ Cell Nucleofector Kit (Lonza) using a Nucleofector 2b Device (Lonza). After electroporation cells were cultured on Cultrex UltiMatrix coated 6-well plates (100 μg/mL, R&D systems) in CD34^+^ expansion media. After 3 days, ReproTeSR (STEMCELL) was added to culture media for 2 more days. On day 7 cells were cultured in ReproTeSR media only. Media was changed daily. After 2–3 weeks, iPSC colonies were isolated manually and transferred to Cultrex coated 6-well plates containing mTeSR Plus media (STEMCELL). Three subclones were created for each iPS cell line.

### Human induced pluripotent stem cells

Human iPSCs were cultured as previously described (Lancaster and Knoblich, 2014; Daviaud et al., 2023). iPSCs were plated in 6-well tissue culture plates coated with diluted Cultrex UltiMatrix (100 μg/mL, R&D Systems) and maintained in mTeSR Plus Culture Media (STEMCELL), supplemented with rock inhibitor Thiazovivin (2 μM, Millipore). Media was changed daily without rock inhibitor until ready to passage at approximately 70–80% confluence or harvested.

**Figure 1:**
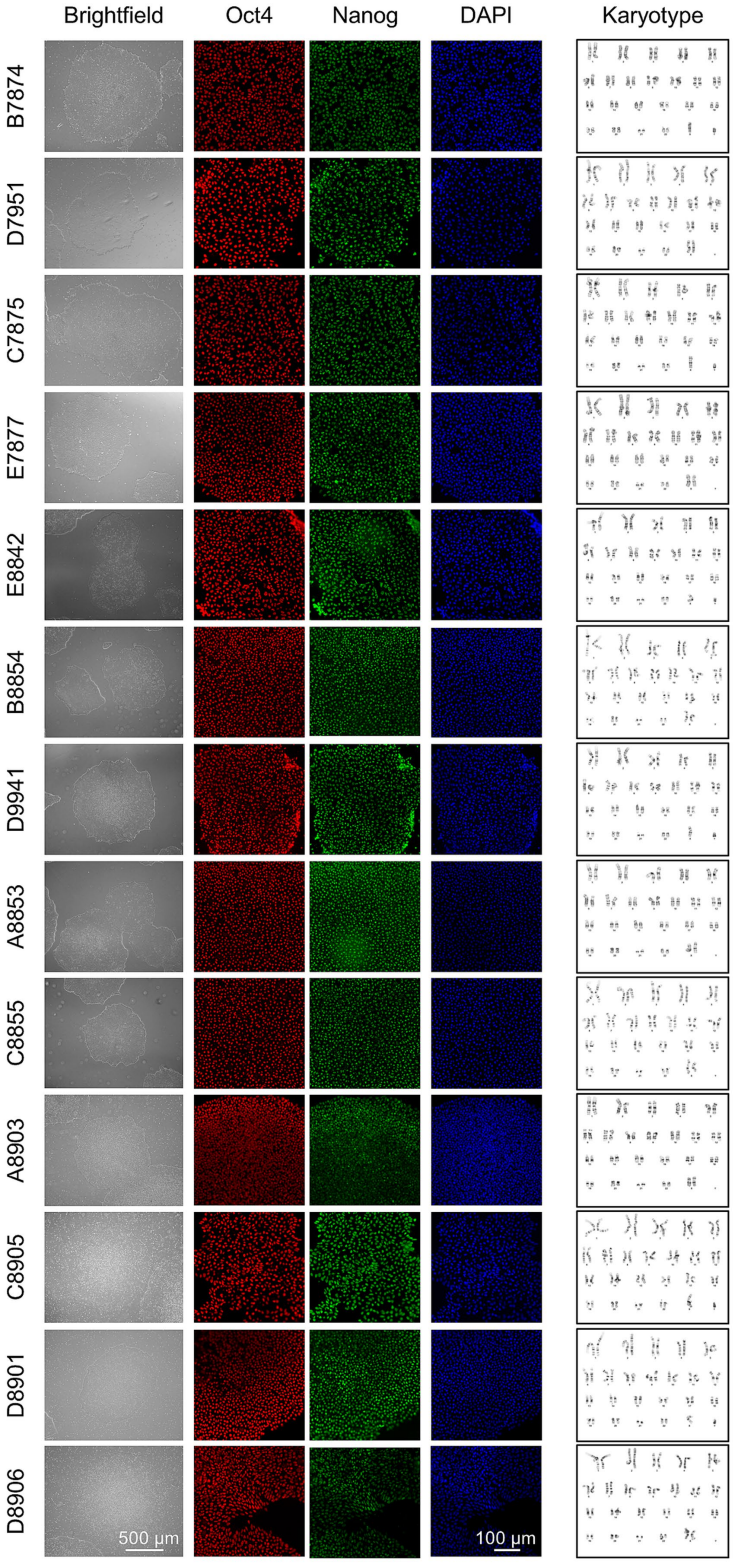
Patient cell lines pluripotency quality control. The pluripotent cell lines established for this work, derived from healthy controls and patients with MS, were tested for pluripotency and underwent quality control analysis. Additional cell lines were previously tested and characterized (Daviaud et al., 2023). Brightfield microscopy was used to assess iPSCs morphology and general health. Oct4 and Nanog immunofluorescence counterstained with DAPI was performed to verify iPSCs pluripotency. Karyotype analysis was performed by G-band testing to confirm genetic stability after reprogramming.

All human pluripotent stem cells were maintained below passage 30 and confirmed negative for mycoplasma using the MycoFluor Mycoplasma Detection Kit (ThermoFisher). iPSCs were regularly evaluated for pluripotency using OCT4, NANOG and SOX2 markers (Figure 1) and were confirmed to be karyotypically normal by G-band testing by a qualified service provider (Cell Line Genetics, Madison, WI, USA) (Figure 1).

### Generation of neural precursor cells

Neural precursor cells (NPCs) were produced following a protocol previously described (Gunhanlar et al., 2018) with minor modification. Briefly, iPSCs were dissociated with EDTA (0.5 mM, Millipore) for 5–6 min at 37 °C. Embryonic bodies (EBs) were generated by transferring 4,000 cells per well of an ultra-low attachment 96 well plates in mTeSR Plus supplemented with Thiazovivin (2 μM, Millipore). After 2 days, the medium was changed to neural induction media (StemDiff, STEMCELL) and EBs were cultured for another 4 days. On day 7, EBs were slightly dissociated by mechanical trituration and cultured on Cultrex UltiMatrix (100 μg/mL, R&D systems) coated plates in neural induction medium (StemDiff, STEMCELL) for 7 days. At d15 the medium was switched to NPC medium consisting of DMEM/F12, 1% N2 supplement (ThermoFisher), 2% B27 supplement without RA (ThermoFisher), 20 ng/mL epithelial growth factor (Peprotech), 10 ng/mL basic fibroblast growth factor (Peprotech) and 1% penicillin/streptomycin (ThermoFisher). At d15, cells were considered pre-NPCs and could be passaged and cryopreserved at confluence. From passage 3, cells were considered NPCs and were used for histologic analysis and for neural differentiation.

Neural differentiation was achieved by mitogen withdrawal. NPCs were cultured for 10 days on Cultrex UltiMatrix coated plates with differentiation media consisting of DMEM/F12, 1% N2 supplement and 2% B27 with RA supplement (ThermoFisher) and were then fixed and analyzed by immunostaining.

### Generation of human cerebral organoids

C-organoids were generated from human iPSCs and processed for analysis as described (Lancaster and Knoblich, 2014; Daviaud et al., 2023) with minor modifications. iPSCs were washed with Dulbecco’s phosphate-buffered saline (DPBS, ThermoFisher) and dissociated with EDTA 0.5 mM (Millipore). A total of 8 × 103 cells were seeded into each well of an ultra-low attachment 96-well plate (Corning) to form embryoid bodies (EBs) in mTeSR Plus medium supplemented with 4 μM of Thiazovivin (Millipore) for the first 2 days. The medium was changed every other day to the same medium without Thiazovivin for another 2–3 days. After 4–5 days of culture or when EBs reached ~500–600 μm in diameter and the surface tissue began to brighten, EBs were cultured in neural induction medium (StemDiff, STEMCELL). After neuroepithelium emergence (typically at ~ day 9–10), embryoid bodies were embedded in 15 μL Cultrex UltiMatrix droplets and cultured in 6 well plates containing c-organoid differentiation medium consisting of 1:1 DMEM-F12 and Neurobasal medium (Gibco), with addition of 0.5% N2 supplement (Life Technologies), 0.5% ml MEM-NEAA (Gibco), 1% Glutamax (Gibco), 1% B27 supplement without vitamin A (Life Technologies), 0.1 μM of 2-mercaptoethanol (Millipore), 2.6 μg/mL insulin (Sigma Aldrich) in static culture for 4 days. Organoids were cultured in c-organoid differentiation medium supplemented with vitamin A on an orbital shaker (CO2 Resistant Shakers, ThermoFisher) at 80 rpm. Organoids were cultured up to 150 days. Analyses were performed at D42 and D120. D42 corresponds to an early stage when cortical structures, neural stem cells, NPCs, and neuroblasts are well-formed, but mature astrocytes and myelinating oligodendrocytes are not yet present. D120 represents a later stage when mature neurons and glial cells, including myelinating oligodendrocytes, are detectable.

### Generation of human spinal cord organoids

Spinal cord organoids were generated from human iPSCs and processed for analysis as described elsewhere (Lee et al., 2022; Xue et al., 2023) with minor modifications. iPSCs were washed with DPBS (ThermoFisher) and dissociated with EDTA 0.5 mM (Millipore). A total of 8 × 10^3^ cells were seeded into each well of an ultra-low attachment 96-well plate (Corning) to form embryoid bodies (EBs) in mTeSR Plus medium supplemented with 4 μM of Thiazovivin (Millipore) for the first 2 days. On day 3, the totality of the culture media was removed and replaced with a differentiation media consisting of 1:1 DMEM-F12 and Neurobasal medium (Gibco), with addition of 0.5% N2 supplement (Life Technologies), 0.5% ml MEM-NEAA (Gibco), 1% Glutamax (Gibco), 1% B27 supplement without vitamin A (Life Technologies), 0.1 μM of 2-mercaptoethanol (Millipore), 2.6 μg/mL insulin (Sigma Aldrich), 10 μM of SB431542 (STEMCELL), 2 μM of CHIR99021 (STEMCELL) and 0.5 μM of LDN-193189 (STEMCELL). Organoids were kept in culture for 8 days with half media change every other day. On day 11, organoids were embedded in 15 μL Cultrex UltiMatrix droplets and cultured in 6 well plates containing differentiation medium supplemented with vitamin A and 15 ng/mL of BMP4 (PEPROTECH) on an orbital shaker (CO2 Resistant Shakers, ThermoFisher) at 80 rpm. On day 16, media was changed to a differentiation media supplemented with 10 ng/mL BDNF and 10 ng/mL GDNF (PEPROTECH). Organoids were cultured for 64 days. At this stage, neurons, astrocytes, and motor neurons are clearly detectable, consistent with established spinal cord organoid differentiation protocols.

### Histological analysis

At D42 and D120-150 days of culture, c-organoids were washed in DPBS and fixed in 4% PFA (ThermoFisher) for 20 min at 4 °C. After three washes with DPBS, organoids were cryoprotected in 30% sucrose overnight at 4 °C followed by snap freezing in OCT compound (ThermoFisher) and stored at −20 °C. Cryosections of organoids were cut at 15 μm thickness using a cryostat (Leica CM 1950) and mounted on microscope slides (Histobond^+^, VWR).

For immunofluorescence, slides were thawed to room temperature before being outlined with a PAP pen (Millipore) to create a hydrophobic barrier. Slides were washed and permeabilized with DPBS supplemented with 0.1% Triton X-100 (Millipore Sigma). Non-specific binding sites were blocked with DPBS supplemented with 0.1% Tween 20, 4% Bovine Serum Albumin (ThermoFisher) and 10% Normal Goat Serum (ThermoFisher) for 1 h at RT. Slides were then incubated overnight at 4 °C with the following primary antibodies diluted in blocking solution: mouse anti-APC (1:100, Calbiochem), mouse anti-ChAT (1:200, ThermoFisher), rat anti-CTIP2 (1:500, Abcam), guinea pig anti-DCX (1:500, Millipore), Rabbit anti-E2F1 (1:400, ThermoFisher), mouse anti-EOMES (1:100, ThermoFisher), mouse anti-GAD67 (1:200, Abcam), mouse anti-GFAP (1:500, Novus Biologicals), mouse anti-Ki67 (1:400, Millipore), chicken anti-MBP (1:500, ThermoFisher), mouse anti-Nanog (1:400, Abcam), mouse anti-O4 (1:200, R&D systems), rabbit anti-Oct4 (1:400, Abcam), rabbit anti-Olig2 (1:200, Abcam), rabbit anti-p16 (1:200, ThermoFisher), rabbit anti-p21 (1:200, ThermoFisher), rabbit anti-p27 (1:400, ThermoFisher), rabbit anti-p57 (1,400, ThermoFisher), mouse anti-Pax6 (1,100, Abcam), rabbit anti-PAK1 (1,100, ThermoFisher), mouse anti-SOX2 (1:400, Abcam), rabbit anti-TBR1 (1,400, Abcam), rabbit anti-vGluT1 (1,200, Abcam). After washing, slides were incubated with appropriate Alexa-coupled secondary antibodies (ThermoFisher) diluted in blocking solution for 1 h at RT and counterstained with DAPI, before mounting with Fluoromount Aqueous Mounting Medium (Millipore).

Immunofluorescence images were collected using a fluorescence microscope (Zeiss Imager M2) or a confocal fluorescence microscope (Zeiss LSM 510) and processed using Zen software and Fiji software (Schindelin et al., 2012).

### Experimental design and statistical analysis

Organoids were derived from 20 patient iPSC lines, with a minimum of three lines per MS subtype. Three independent subclones were generated for each line. For each clone, 2–4 independent differentiation experiments (batches) were performed on separate days using independently prepared media. Each differentiation experiment generated 1–3 organoids. Multiple images (2–4) were acquired per organoid and quantified using Fiji software (Schindelin et al., 2012) and averaged.

For quantification, regions of interest (ROIs) were defined as radial columns spanning all cortical layers near the organoid surface. ROIs were positioned according to consistent anatomical criteria across all samples. For cell number analyses, marker-positive cells within each ROI were manually counted. Only cells showing clear co-localization of marker signal with a DAPI-positive nucleus were considered positive. For percentage analyses, the number of marker-positive cells was normalized to the total number of DAPI-positive nuclei within the same ROI and expressed as a percentage. For fluorescence intensity analyses, absolute fluorescence intensity (arbitrary units, A.U.) was measured within the same ROIs using identical acquisition and analysis parameters across all samples. Fluorescence intensity values were normalized to the total DAPI fluorescence intensity within each ROI to account for differences in cell density. No background subtraction or *post hoc* exclusion of artefactual staining was performed.

Statistical analyses were performed using GraphPad Prism 11 (GraphPad Software). Data with a hierarchical structure (technical replicates nested within patient-derived iPSC lines and grouped by clinical condition) were analyzed using a nested one-way ANOVA, with condition treated as a fixed effect and patient as a random effect nested within condition. When a significant overall effect was detected, Tukey’s multiple comparison test was applied. For comparisons involving single measurements per subject, differences between groups were analyzed using one-way ANOVA. Residual normality was assessed using the Shapiro–Wilk test. A chi-squared test was used to analyze frequency distributions where appropriate. Data are presented as mean ± SEM unless otherwise specified. Results were considered statistically significant when *p* < 0.05.

## Results

### Cerebral organoid derived from patients with MS develop and mature over time

MS is a heterogeneous disease whose course and severity can vary greatly from patient to patient. To best capture the diversity/variability of the disease, we decided to generate iPS cell lines derived from 10 male and 10 female subjects (Table 1), including 6 healthy controls and 14 MS patients. The age of the patients ranged from 23 to 67 years old with no significant differences between healthy controls and the different MS groups (One-Way ANOVA, *p* = 0.3450) (Table 2).

Each patient iPS cell line exhibited a normal phenotype in culture. Expression of pluripotency markers, such as Oct4 and Nanog were confirmed by immunofluorescence, and each cell line had a normal Karyotype (Figure 1).

**Figure 2:**
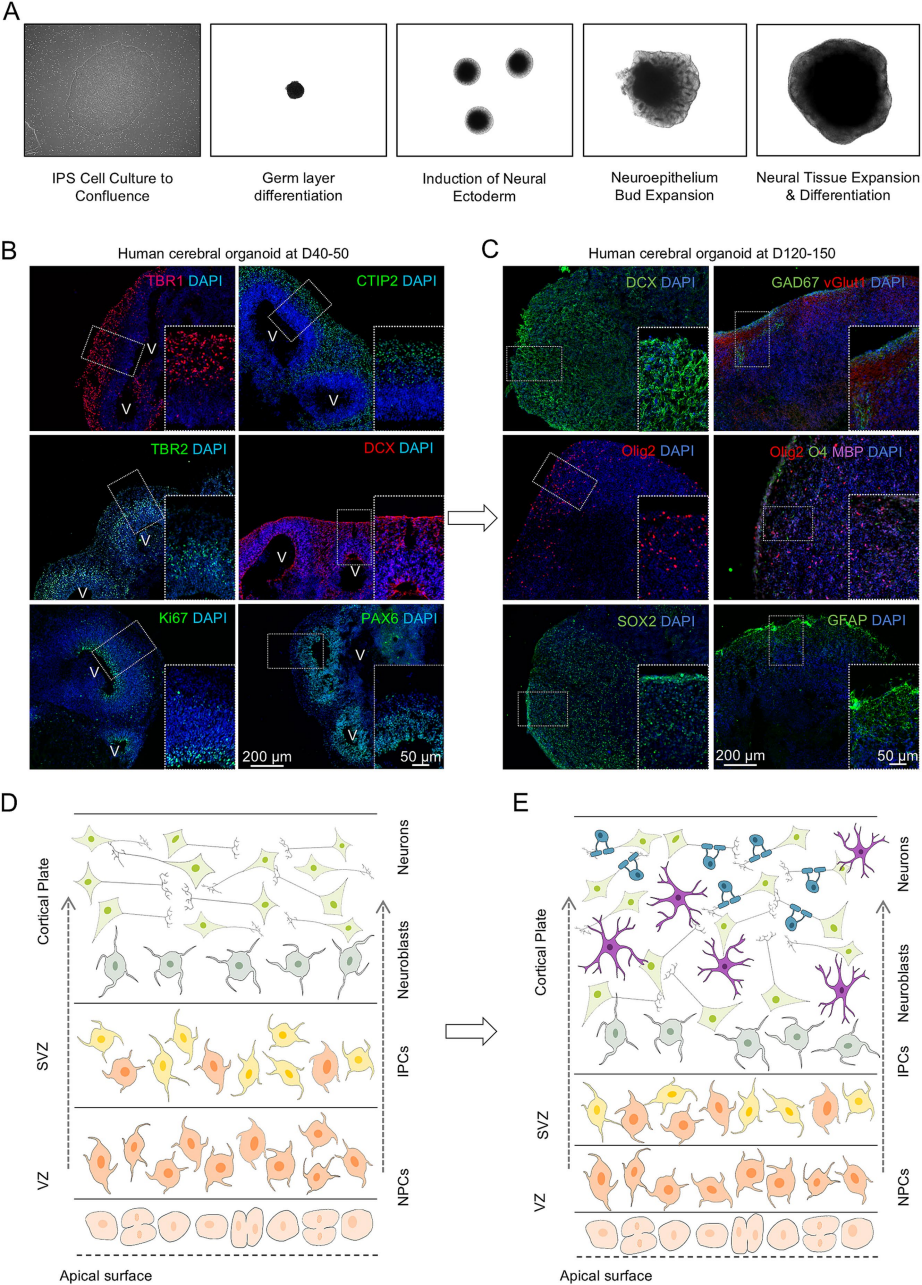
Cerebral organoids derived from patients with MS develop and mature over time. **(A)** Pictures of IPS cells derivation into cerebral organoid using brightfield microscopy. **(B)** Immunofluorescence of human cerebral organoids at D40-50 for the major cell types detected: Ki67^+^ proliferating cells, SOX2^+^ neural precursors, TBR2^+^ intermediate progenitors, DCX^+^ neuroblasts, TBR1^+^ and TBR2^+^ mature cortical neurons. V: Ventricles. Insets show higher magnification views of representative ZOIs (200 × 300 μm). **(C)** Immunofluorescence of human cerebral organoids at D120-150 for SOX2^+^ neural precursors, GFAP^+^ astrocytes, Olig2^+^ oligodendrocytic cells, DCX^+^ neuroblasts, GAD67^+^ and vGlut1^+^ GABAergic and glutamatergic neurons respectively and Olig2^+^O4^+^MBP^+^ myelinating oligodendrocytes. Insets show higher magnification views of representative ZOIs (200 × 300 μm). **(D)** Schematic representation of the cortical structures found in cerebral organoids at D40-50 including the ventricular zone containing the stem cell pool and most proliferating cells, the subventricular zone (SVZ) containing mostly iPCs, the outer SVZ (oSVZ) and the cortical plate (CP) containing neuroblasts and mature neurons. **(E)** Schematic representation of the structures found in cerebral organoids at D120-150. At this point there is no clear distinction between cortical layers. The rim of the organoids contains stem cells but mostly mature neurons, astrocytes, and myelinating oligodendrocytes.

C-organoids were generated using a previously described protocol (Lancaster and Knoblich, 2014; Daviaud et al., 2023) (Figure 2A). After 40–50 days *in vitro*, c-organoids exhibited immature cortical structures consisting of the ventricle aligned with proliferating cells, surrounded by the ventricular zone (VZ) containing the SOX2^+^ stem cell pool, the subventricular zone (SVZ) containing TBR2^+^ intermediate progenitors and DCX^+^ neuroblasts, and the cortical plate (CP) containing mature neurons (Figures 2B,D). After 120–150 days in vitro, c-organoids continued to mature, and exhibited more developed neurons, such as GABAergic and Glutamatergic neurons, but also myelinating oligodendrocytes and astrocytes (Figures 2C,E).

### CDKi involved in MS organoids appears to be restricted to p21

**Figure 3:**
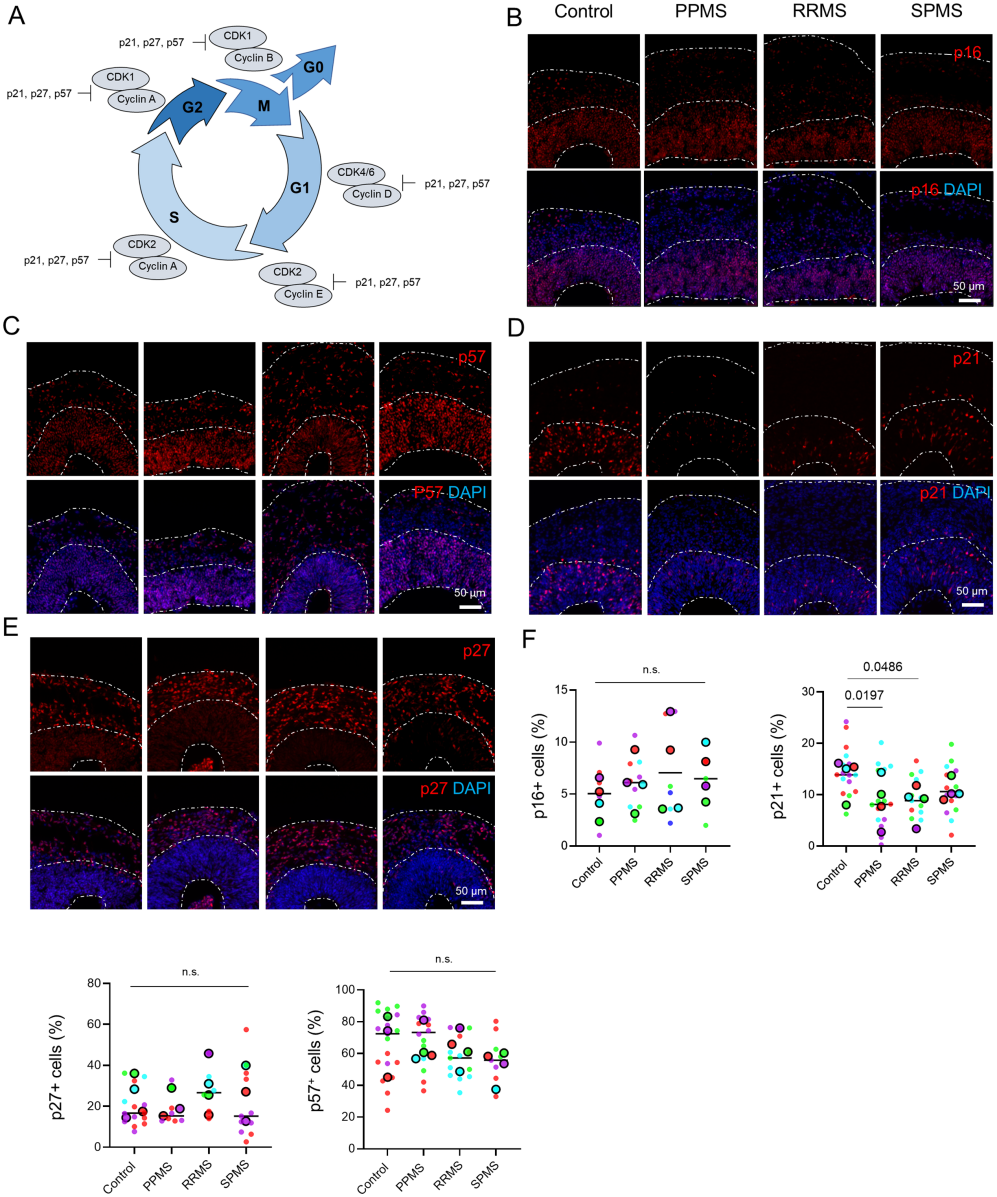
CDKi involved in MS organoids appears to be restricted to p21. **(A)** Schematic representation of the cell cycle and the involvement of the various CDK/cyclin complexes and the different CDK inhibitors. **(B)** Representative images of an immunofluorescence against the CDKi p16 in c-organoids at D42 in the different conditions. No ectopic location or important change in p16 expression were observed. **(C)** Representative images of an immunofluorescence against the CDKi p57 in c-organoids at D42 in the different conditions. p57 was evenly distributed among the layers of c-organoids. No ectopic location or important change in p57 expression were observed. **(D)** Representative images of an immunofluorescence against the CDKi p21 in c-organoids at D42 in the different conditions. p21 was mostly expressed in the lower layers (SVZ and VZ) of c-organoids. No ectopic location was detected, however, an important decrease of p21 expression was observed, especially in PPMS. **(E)** Representative images of an immunofluorescence against the CDKi p27 in c-organoids at D42 in the different conditions. p27 was mostly expressed in the outer layers of c-organoids (CP). No ectopic location or important change in p27 expression were observed. **(F)** Quantification of p16, p27, p57, and p21 expression. Statistical analysis was performed using a nested one-way ANOVA (p16: *p* = 0.7402; p27: *p* = 0.6003; p57: *p* = 0.1491; p21: *p* = 0.0188). Cortical regions were cropped from the original images to include all cortical layers (VZ, SVZ, and CP). Data are presented as superplots: small dots represent individual technical replicates, while large dots represent the mean value for each patient. Horizontal lines indicate the grand mean of the conditions.

CDKi are proteins that bind to and inhibit the activity of CDKs, thereby controlling the cell cycle. Two major classes of CDKi have been identified. The INKs family includes p15, p16, p18 and p19 which bind to and inhibit the activities of CDK4 and CDK6. The CIP/Kips family includes p21, p27, p28 and p57 which can bind to and inhibit the activities of a wide range of CDK-cyclin complexes (Besson et al., 2008) (Figure 3A).

We previously highlighted the involvement of p21 in a c-organoid model of MS (Daviaud et al., 2023). However, we did not evaluate other CDKi expressions in MS organoids compared to controls. Interestingly, p16 expression is increased in PPMS neural stem cells leading to senescence which could contribute to limited remyelination (Nicaise et al., 2019). p27 ensures cell cycle arrest and subsequent differentiation of oligodendrocyte precursor cells (OPCs) in mature oligodendrocytes (Casaccia-Bonnefil et al., 1997) and has also been described as a positive regulator of Schwann cell differentiation *in vitro* (Li et al., 2011). p57 regulates the number of divisions in OPCs before the onset of differentiation and can inhibit oligodendrocyte and Schwann cell differentiation (Nicaise et al., 2019). Thus, abnormalities in any of these CDKi may impact myelin function.

We performed an immunofluorescence at D42 for p16, p21, p27 and p57 in organoids from patients with MS and healthy controls (Figures 3B–E). No ectopic location or significant expression difference was observed for p16, p27 and p57, while a decreased expression of p21 was found in MS organoids, especially in PPMS (Figures 3B–E). Quantification revealed no significant difference for p16 (Nested one-Way ANOVA, *p* = 0.7402), p27 (Nested one-Way ANOVA, *p* = 0.6003), p57 (Nested one-Way ANOVA, *p* = 0.1491). In contrast, a significant overall group effect was observed for p21 expression (Nested one-way ANOVA, *p* = 0.0188) with reduced p21 expression in PPMS (*p* = 0.0197) and RRMS (*p* = 0.0486) compared with controls (Figure 3F).

### The CDKi involved in MS NPC in vitro appears to be limited to the cell cycle inhibitor p16

**Figure 4:**
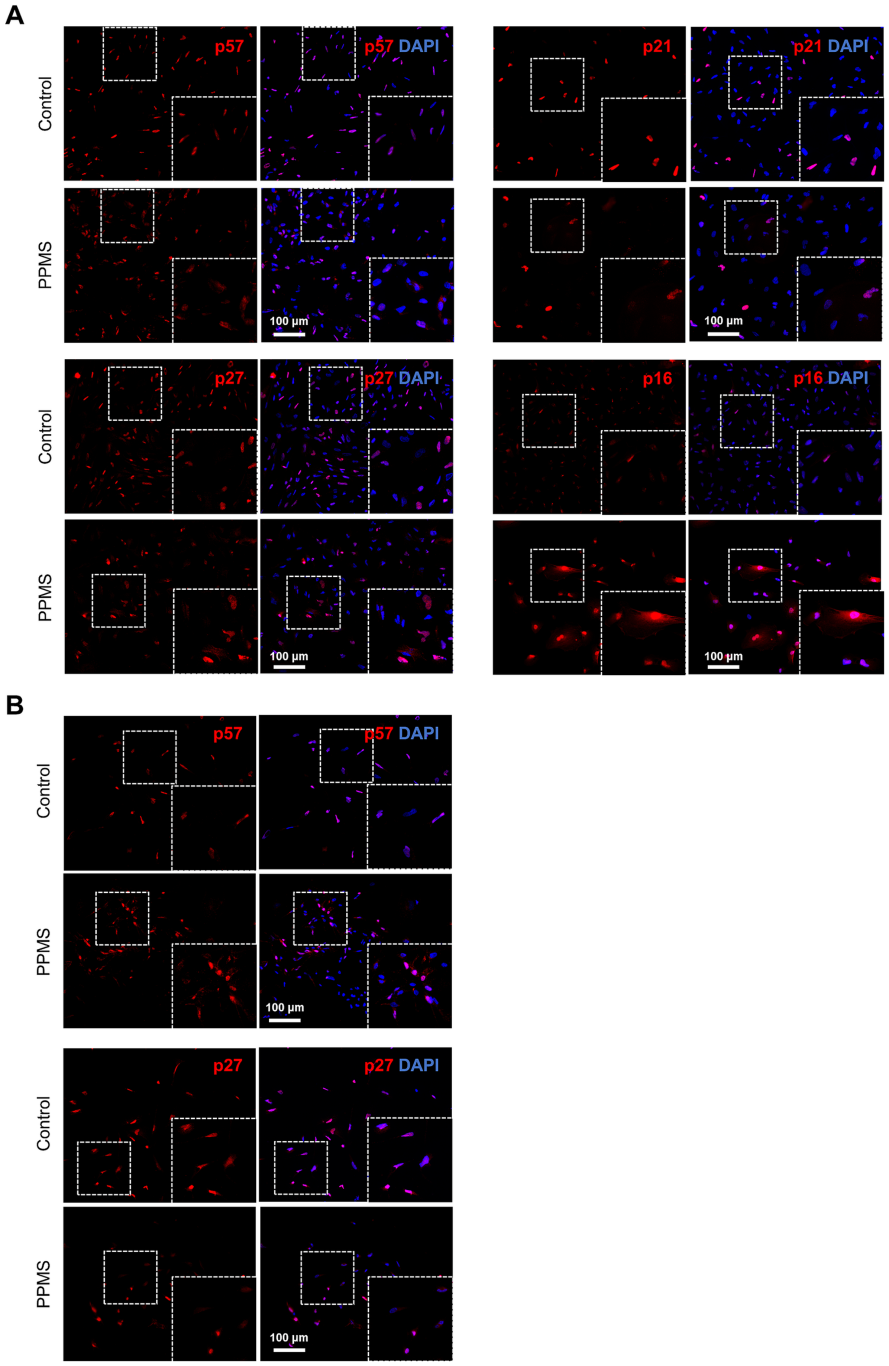
iPSC derived NPC and neural cell from PPMS exhibit p16 induced senescence. **(A)** Representative images of an immunofluorescence performed on iPS-derived NPC in expansion for p57, p21, p27, and p16, in control and PPMS samples. Only p16 expression was different between the two conditions. **(B)** Representative images of an immunofluorescence performed on iPS-derived NPC, 8 days after mitogens withdrawal for p57 and p27 in control and PPMS samples. Insets show higher magnification views of representative ZOIs (150 × 150 μm).

To confirm the results observed in c-organoids in a simpler model, we decided to perform immunofluorescence for the CDKi p16, p21, p27 and p57 on iPS-derived NPCs. We performed these analyses on 2 conditions: NPC in expansion and NPC after 10 days of neural differentiation (Figure 4). In proliferating PPMS NPCs, p21 and p27 expression were detected in both PPMS and control conditions with no apparent differences based on qualitative assessment. In contrast, there appeared to be a visual difference in p16 expression and associated cellular morphology in PPMS NPCs compared to controls, consistent with previous findings (Nicaise et al., 2019) (Figure 4A). A potential translocation of p57 from the nucleus to the cytoplasm was noted in PPMS NPCs compared to controls under both proliferative and differentiation conditions (Figure 4A). Similarly in differentiation conditions, p27 expression was detected in PPMS and control cells with no apparent change (Figure 4B). Quantitative analyses would be required to formally assess differences in expression levels or subcellular localization.

### P21 regulators E2F1 and PAK1 are involved in MS pathogenesis

**Figure 5:**
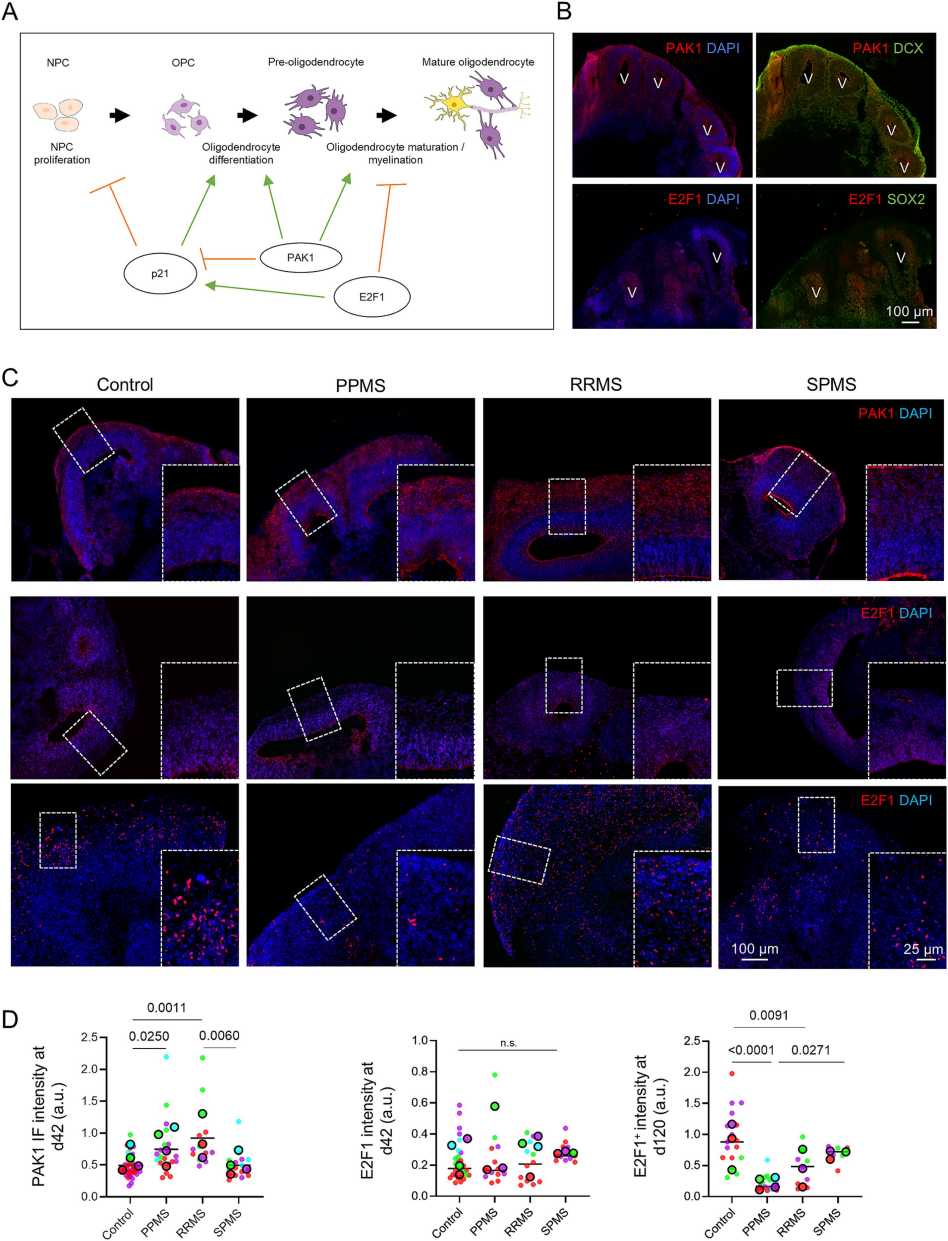
P21 regulators E2F1 and PAK1 are involved in MS pathogenesis. **(A)** Schematic representation of the involvement of PAK1 and E2F1 in p21 regulation, but also in oligodendrocyte differentiation, maturation, and myelination capacity. **(B)** Immunofluorescence for PAK1 co-stained with neuroblast marker DCX and E2F1 co-stained with stem cell marker SOX2. **(C)** Representative images of an immunofluorescence for PAK1 and E2F1 in organoids at D42 and E2F1 at D120 in control, PPMS, RRMS, and SPMS samples. **(D)** Quantifications for PAK1 at D42 (Nested one-way ANOVA, *p* = 0.0004), E2F1 at D42 (Nested one-way ANOVA, *p* = 0.5999), and E2F1 in c-organoids at D120 (Nested one-way ANOVA, *p* < 0.0001). Insets show higher magnification views of representative ZOIs (200 × 300 μm). Data are presented as Superplots: small dots represent individual technical replicates, while large dots represent the mean value for each patient. Horizontal lines indicate the grand mean of the conditions.

We found that CDKi p21 is significantly decreased in MS organoids, especially in PPMS. As p21 is necessary for oligodendrocyte differentiation, we analyzed PAK1 and E2F1 expressions. PAK1 is likewise involved at multiple stages: it promotes OPC differentiation into pre-oligodendrocytes and supports maturation into myelinating oligodendrocytes (Brown et al., 2021), while also regulating cortical development by enhancing neural progenitor proliferation and neuronal migration (Pan et al., 2015). Notably, PAK1 silencing has been shown to increase p21 expression, suggesting a functional interaction between these pathways (Wang et al., 2018). In parallel, E2F1 negatively regulates oligodendrocyte maturation, and its transcript levels decrease as OPCs differentiate into oligodendrocytes (Magri et al., 2014). E2F1 is also required for Ras-mediated activation of the p21 promoter (Gartel et al., 2000), highlighting the complex interplay between cell cycle regulators and oligodendrocyte differentiation programs (Figure 5A).

We first determined the localization of E2F1 and PAK1 expression in c-organoids at D42 by immunofluorescence. PAK1 was mainly expressed in the outer layers of the organoid cortical structure and the cortical plate, suggesting that PAK1 was mainly expressed in mature cells, such as neuroblasts, astrocytes or oligodendrocytes (Figure 5B). E2F1 was mainly expressed in the inner layer of the cortical structure, especially in the ventricular zone, which indicates that E2F1 was mainly expressed in the progenitor cells, such as NPCs (Figure 5B).

We then performed an immunofluorescence for PAK1 and E2F1 in c-organoids from patients with MS compared to organoids from healthy controls (Figure 5C). At D42, very low to no E2F1 expression was detected, and quantification revealed no significant differences between groups (Nested one-way ANOVA, *p* = 0.5999). In contrast, at D120, strong E2F1 staining was observed, consistent with its reported role in more mature neural contexts, including neuronal and glial differentiation. A significant overall group effect was observed for E2F1 expression (Nested one-way ANOVA, *p* < 0.0001) with reduced E2F1 expression in PPMS organoids compared with controls (*p* < 0.0001) and RRMS (*p* = 0.0091), while no significant difference was observed compared with SPMS (*p* = 0.3450).

At D42, a significant overall difference in PAK1 expression was also detected (Nested one-way ANOVA, *p* = 0.0004) with increased PAK1 expression in PPMS (*p* = 0.0250) and RRMS (*p* = 0.0011) compared with controls (Figure 5D).

These results suggest that the dysregulation of p21 may be induced or associated with changes in E2F1 and PAK1 expression in the c-organoid model of MS.

### Oligodendrocyte maturation and myelination capacity is reduced in MS organoids

**Figure 6:**
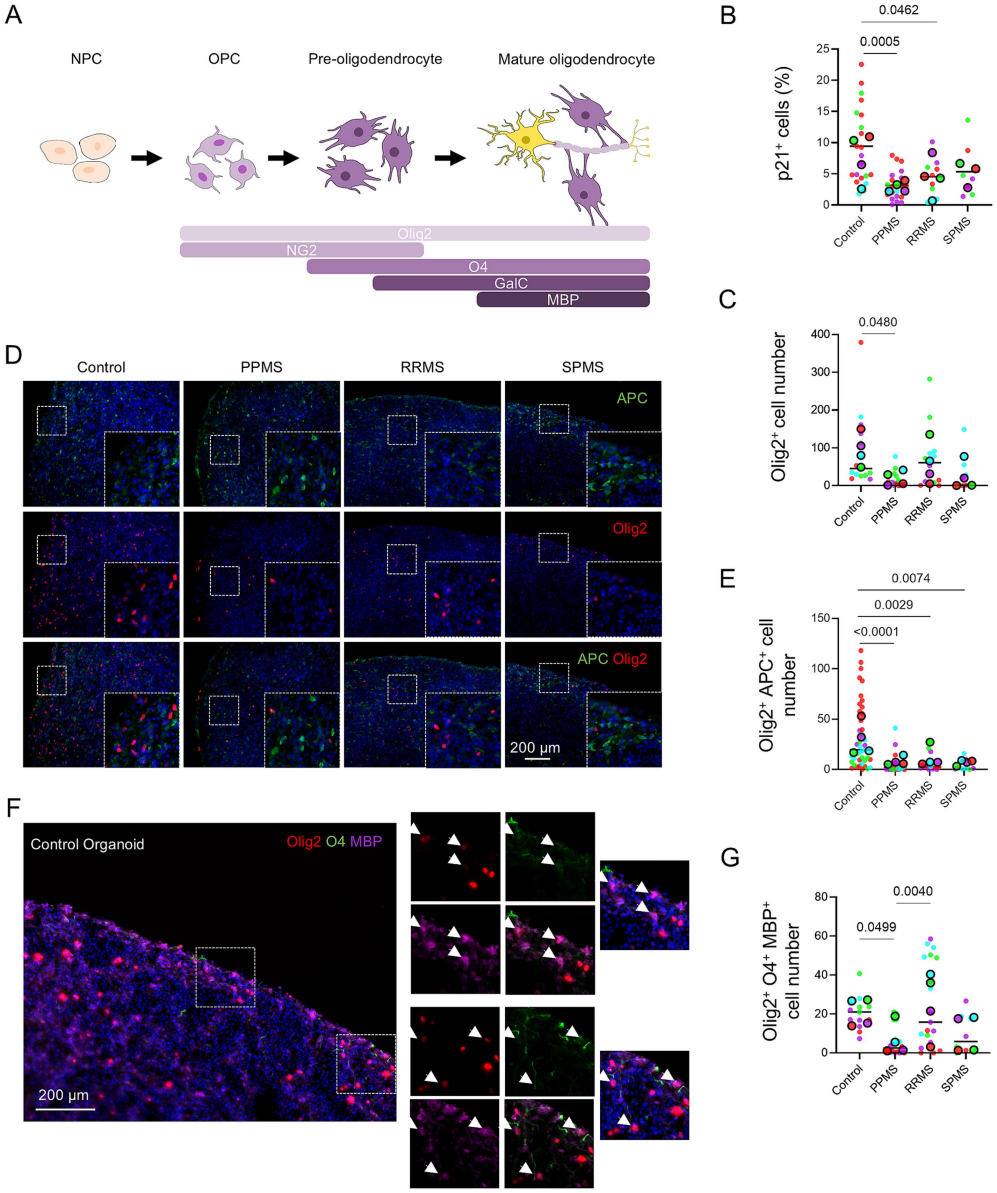
Oligodendrocyte maturation and myelination capacity is reduced in MS organoids at D120. **(A)** Schematic representation of the different markers expressed during the differentiation of NPCs into OPCs and their maturation into pre-oligodendrocytes and mature myelinating oligodendrocytes. **(B)** Quantification of p21^+^ cells in c-organoids at D120 highlighting a significant change in PPMS organoids (Nested one-way ANOVA, *p* = 0.0009). **(C)** Quantification of Olig2^+^ cells in c-organoids at D120, showing a significant difference in PPMS organoids (Nested one-way ANOVA, *p* = 0.0410). **(D)** Representative immunofluorescence images showing Olig2, a marker of the oligodendrocyte lineage, and APC, which labels mature oligodendrocytes and can also be expressed in astrocytes; therefore, Olig2^+^/APC^+^ cells were identified as mature oligodendrocytes. Insets show higher magnification views of representative ZOIs (100 × 100 μm). **(E)** Quantification of Olig2^+^APC^+^ double positive cells in c-organoids at D120. A significant difference was detected (Nested one-way ANOVA, *p* < 0.0001). **(F)** Representative images of an immunofluorescence for Olig2, O4, and MBP in control c-organoids at D120. The orange squares represent the zone of interest highlighted on the right panel. White arrowheads indicate triple positive cells. Colocalization of those three markers indicates the presence of myelinating oligodendrocytes. Insets show higher magnification views of representative ZOIs (100 × 100 μm). **(G)** Quantification of Olig2^+^O4^+^MBP^+^ triple positive cells in c-organoids at D120. A significant difference was detected (Nested one-way ANOVA, *p* = 0.0040). Data are presented as Superplots: small dots represent individual technical replicates, while large dots represent the mean value for each patient. Horizontal lines indicate the grand mean of the conditions.

The differentiation of NPCs into mature oligodendrocytes is a multi-step process. NPCs first differentiate into NG2^+^ OPCs, then into O4^+^ pre-oligodendrocytes which mature into GALC^+^/MBP^+^ myelinating oligodendrocytes. During each of these steps, oligodendroglial cells express Olig2 (Figure 6A).

We assessed p21 expression at D120 by immunofluorescence. Quantification revealed a significant overall difference among groups (Nested one-way ANOVA, *p* = 0.0009). Analysis showed a significant decrease in p21 expression in PPMS organoids compared with controls (*p* = 0.0005) and RRMS (*p* = 0.0462) (Figure 6B). This result indicates that the p21 decrease in MS organoids occurs continuously from D28 to D120 and is not a transient effect.

We first studied Olig2 expression in oligodendroglial cell population. Quantification revealed a significant overall difference among groups (Nested one-way ANOVA, *p* = 0.0410). A significant decrease in Olig2^+^ cell number was found in PPMS compared to control (*p* = 0.0480) (Figure 6C). To further assess oligodendrocyte maturation, we performed a double immunofluorescence for the oligodendroglial cell marker Olig2 and the mature oligodendrocyte/astrocyte marker APC (Figure 6D). Double positive cells indicated the presence of mature oligodendrocytes in our organoid model of MS. Quantification indicated a significant overall difference among groups (Nested one-way ANOVA, *p* < 0.0001) with a significantly lower number of Olig2^+^/APC^+^ cells in PPMS (*p* < 0.0001), RRMS (*p* = 0.0029) and SPMS (*p* = 0.0074) organoids compared to control (Figure 6E).

We analyzed the maturation of oligodendrocytes into myelinating oligodendrocytes, by triple immunofluorescence for Olig2, the mature oligodendrocyte marker O4, and the myelinating oligodendrocyte marker MBP (Figure 6F). Triple positive cells were found in control organoids. Quantification highlighted a significant overall difference among groups (Nested one-way ANOVA, *p* = 0.0040), with a significantly lower number of Olig2^+^ O4^+^ MBP^+^ cells in PPMS (*p* = 0.0499) organoids compared to control (Figure 6G).

**Figure 7:**
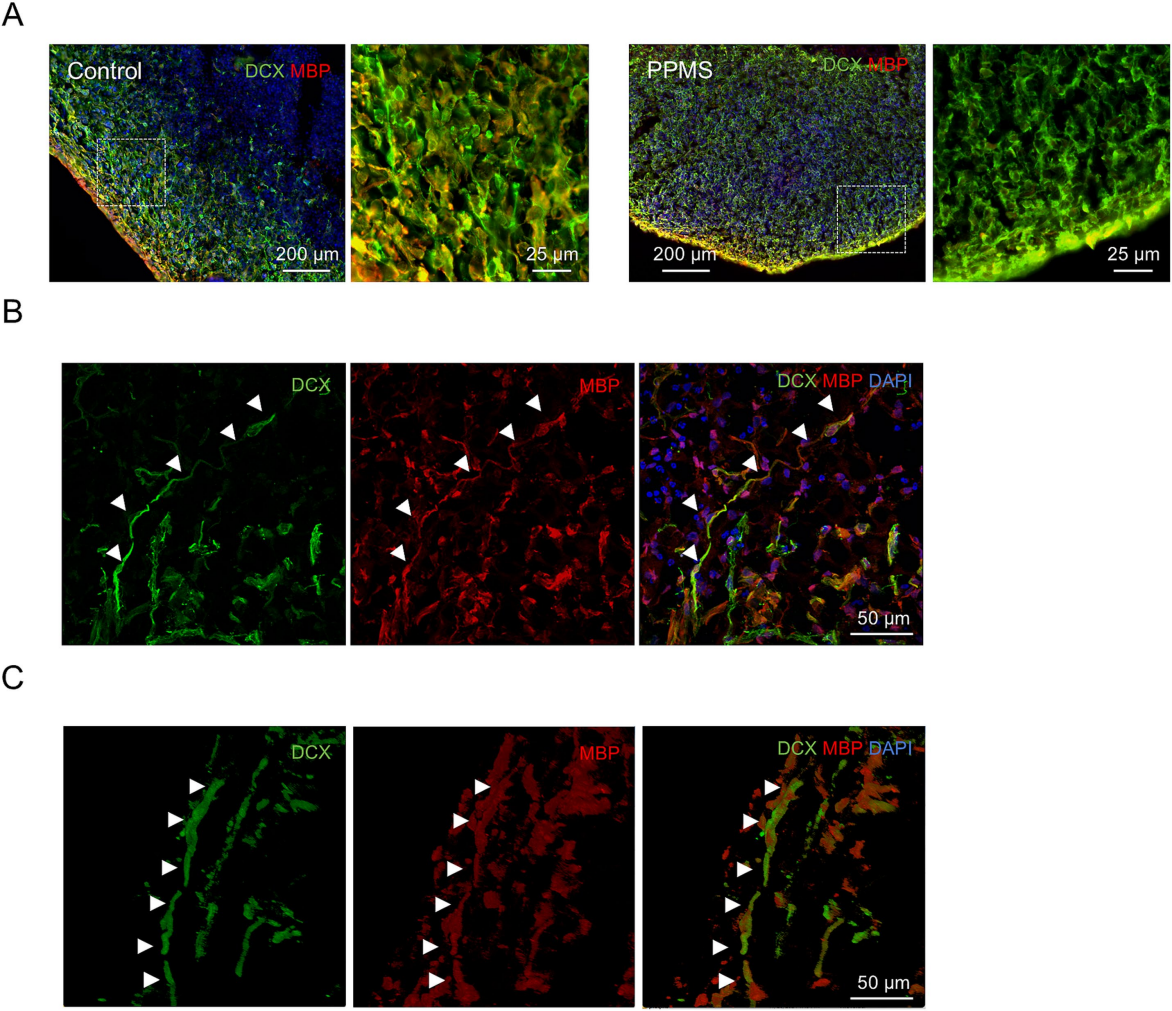
Organoid model of MS contains myelinating neurons. **(A)** Representative images of an immunofluorescence for neuron marker DCX and myelin marker MBP in control and PPMS organoids at D120. Insets show higher magnification views of representative ZOIs (100 × 100 μm). **(B)** Confocal microscopy was performed to confirm colocalization of axons with myelin marker, showing proper myelination. White arrowhead indicates DCX and MBP colocalization. **(C)** 3D reconstruction of stacked images taken by confocal microscopy to further observed myelinated axons in c-organoids. White arrowhead indicates DCX and MBP colocalization.

Finally, we performed an immunofluorescence for the myelin marker MBP and the neuronal marker DCX to verify the quality of the myelin produced in our organoid at D120-150. In control organoids we observed colocalization of the neuronal marker DCX and the myelin marker MBP, highlighting formation of myelin around the axons. By contrast, minimal to absent colocalization occurred in PPMS organoids (Figure 7A). We used confocal microscopy to further confirm the quality of myelin in control organoids (Figure 7B) and performed 3D reconstruction of stacked images (Figure 7C). In control organoids, we observed absolute colocalization of the neuronal marker DCX and the myelin marker MBP along the axons, indicating myelination of axons in our organoid model.

Taken together with our previously reported D42 data demonstrating reduced oligodendroglial lineage specification and impaired early maturation in MS organoids (Daviaud et al., 2023), these results indicate that the defect in oligodendrocyte differentiation arises early during organoid maturation and persists during long-term culture, ultimately resulting in impaired myelination, which may partially explain the clinical phenotype of patients, especially of those with PPMS. Moreover, these results correlate temporally with our previously observed corresponding decrease in p21 expression.

### Neurons and astrocyte differentiation are also affected in MS organoids

**Figure 8:**
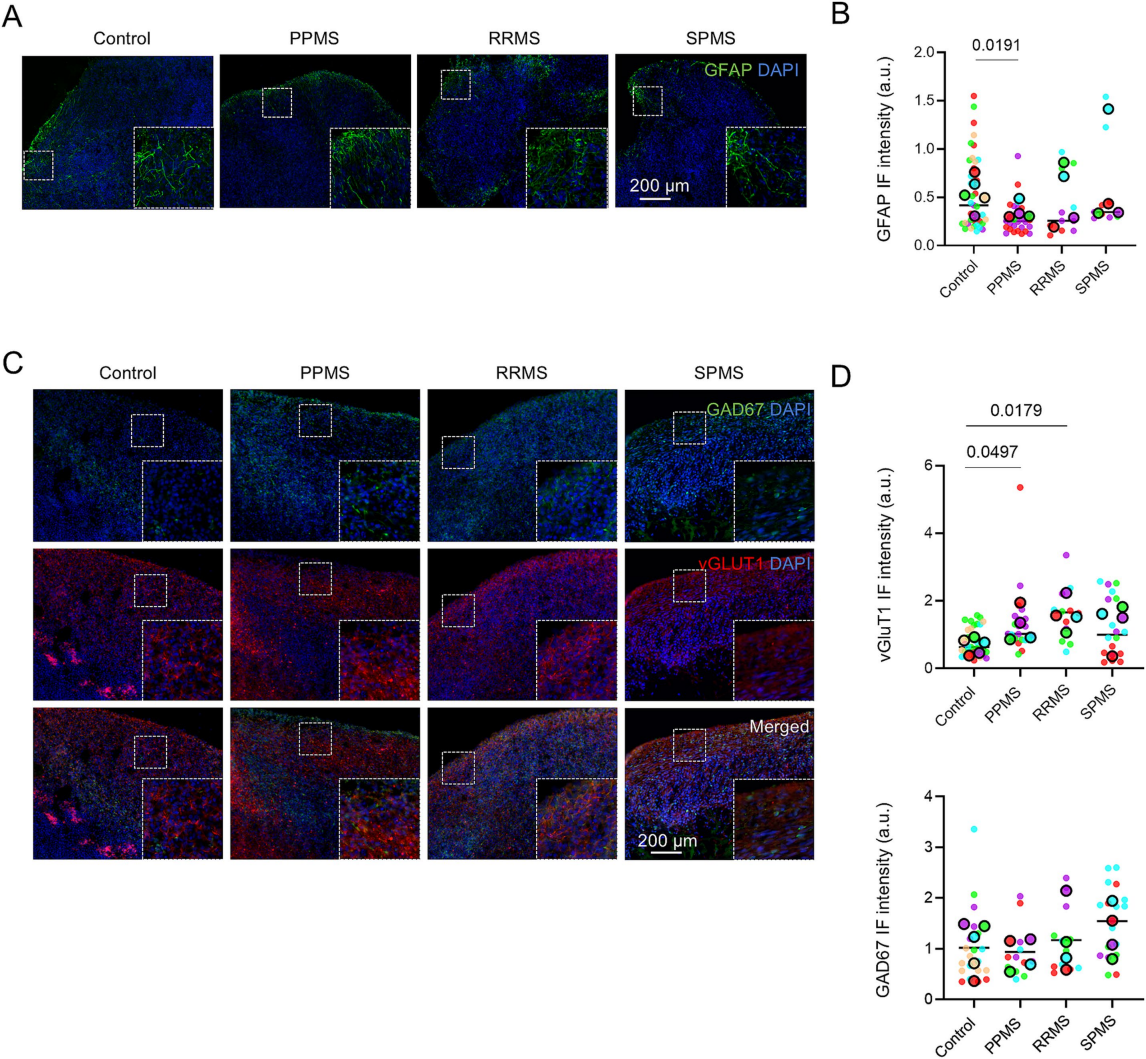
Neuronal and astrocytic differentiation are also affected in MS organoids. **(A)** Representative images of an immunofluorescence for the astrocyte marker GFAP in c-organoids at D120. **(B)** Quantification of GFAP fluorescence intensity in c-organoids at D120. A significant difference was detected (Nested one-way ANOVA, *p* = 0.0206). **(C)** Representative images of an immunofluorescence for the GABAergic neuron marker GAD67 and the glutamatergic neuron marker vGlut1 in c-organoids at D120. **(D)** Quantification of vGlut1 and GAD67 fluorescence intensity in c-organoids at D120. A significant difference was found for vGlut1 (Nested one-way ANOVA, *p* = 0.0117) but not for GAD67 (Nested one-way ANOVA, *p* = 0.0747). Data are presented as Superplots: small dots represent individual technical replicates, while large dots represent the mean value for each patient. Horizontal lines indicate the grand mean of the conditions.

As stated before, at D120, organoids were more mature and exhibited GABAergic and glutamatergic neurons, mature myelinating oligodendrocytes and astrocytes (Figures 2C,E). Astrocytes may contribute to MS pathology by targeting dysregulated immune responses to the CNS and may lead to MS symptoms (Ponath et al., 2018). We identified astrocyte population by immunofluorescence for astrocyte marker GFAP (Figure 8A) and quantification showed a significant overall difference among groups (Nested one-way ANOVA, *p* = 0.0206) with a significant decrease of GFAP expression in PPMS samples compared to control (*p* = 0.0191) (Figure 8B).

We also examined GABAergic and glutamatergic neurons as a dysregulation of GABAergic/glutamatergic neurotransmission is associated with MS symptoms such as fatigue (Arm et al., 2021) or cognitive performance (Huiskamp et al., 2023). We performed an immunofluorescence against glutamatergic neuron marker vGlut1 and GABAergic neuron marker GAD67 (Figure 8C). Quantification revealed a significant overall difference among groups (Nested one-way ANOVA, *p* = 0.0117) with a significant increase of vGlut1 expression in PPMS (*p* = 0.0497) and RRMS (*p* = 0.0179) organoids compared to control (Figure 8D). However, no significant differences in GAD67 expression were observed among MS organoids and controls (Nested one-way ANOVA, *p* = 0.0747) (Figure 8D).

### Spinal cord organoids derived from MS patient exhibit similar phenotype than c-organoids

Spinal cord abnormalities are common in MS and include a variety of pathological processes, such as inflammatory demyelination, neuroaxonal loss and gliosis. Ultimately these changes result in motor weakness associated with gait difficulties, sensory disturbances, as well as bladder and bowel dysfunction (Walker, 2000). To better understand spinal cord pathogenesis in MS, we created SCO from 3 healthy controls as well as 9 MS patients.

**Figure 9:**
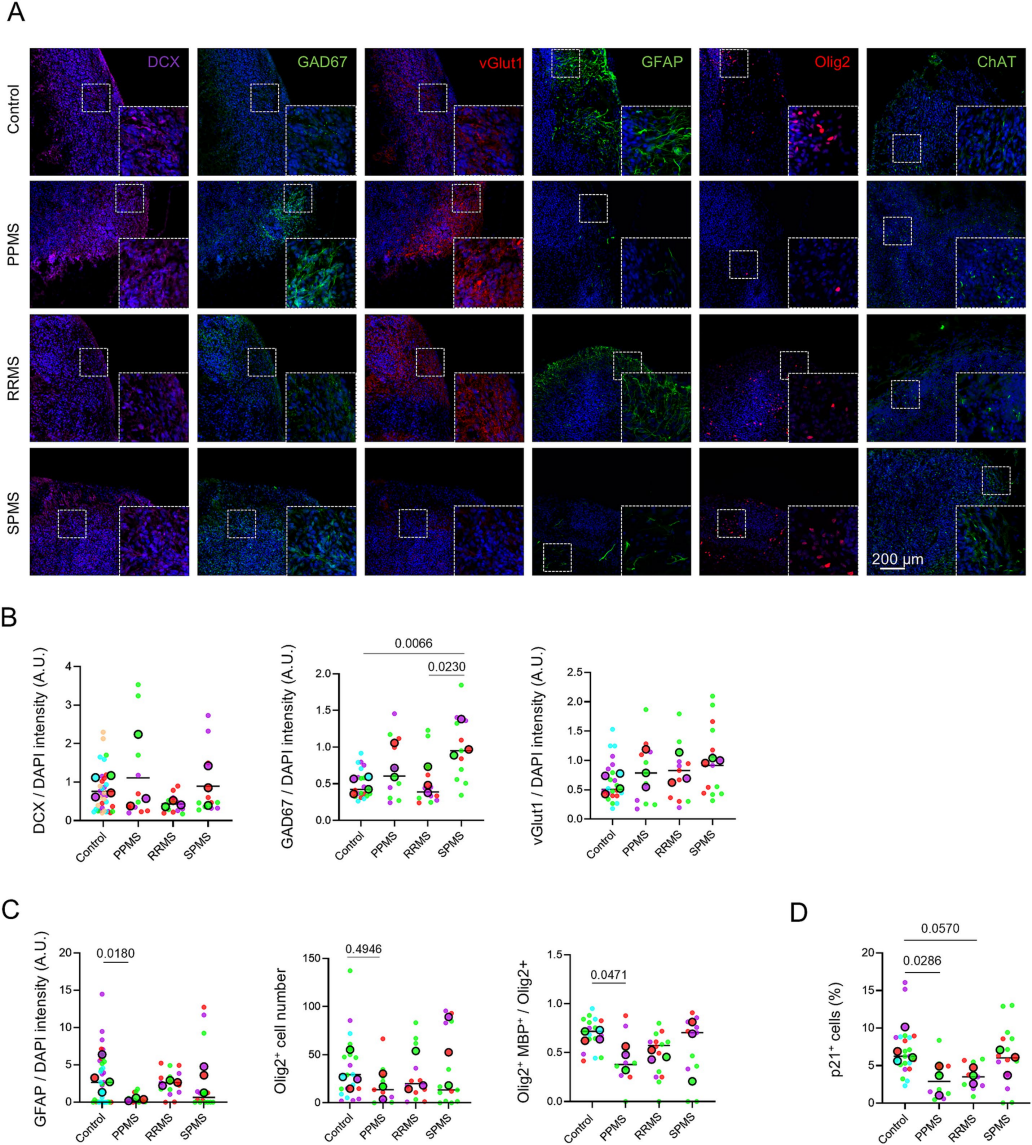
Spinal cord organoids derived from MS patient exhibit similar phenotype than c-organoids. **(A)** Representative images of immunofluorescence for neuroblast marker DCX, GABAergic neuron marker GAD67, glutamatergic neuron marker vGlut1, astrocyte marker GFAP, oligodendrocyte marker Olig2, and motoneuron marker ChAT in spinal cord organoids at D64. Insets show higher magnification views of representative ZOIs (100 × 100 μm). **(B)** Quantification for DCX, GAD67, and vGlut1 fluorescence intensity in spinal cord organoids at D64. Quantification showed no significant difference in DCX expression (Nested one-way ANOVA, *p* = 0.1862) and vGlut1 (Nested one-way ANOVA, *p* = 0.3277), while a significant difference was detected in GAD67 (Nested one-way ANOVA, *p* = 0.0074) expression in MS SCO compared to control. **(C)** Quantification for GFAP fluorescence intensity, Olig2^+^ cell number, and for Olig2^+^MBP^+^ myelinating oligodendrocyte number in spinal cord organoids at D64. Quantifications revealed a significant difference in astrocyte population (Nested one-way ANOVA, *p* = 0.0326). No significant difference was observed for Olig2^+^ cells (Nested one-way ANOVA, *p* = 0.5619). A significant reduction in myelinating oligodendrocyte population (Nested one-way ANOVA, *p* = 0.0416) expression in PPMS SCO compared to control. **(D)** Quantification for p21^+^ cells showed a significant decrease in p21 expression (Kruskal-Wallis, *p* = 0.0058) in PPMS SCOs compared to control while only a slight non-significant decrease was detected in RRMS samples. Data are presented as Superplots: small dots represent individual technical replicates, while large dots represent the mean value for each patient. Horizontal lines indicate the grand mean of the conditions.

The obtained organoids were patterned using WNT activation and BMP signaling to posteriorize neural tissue and suppress cortical fate, and they formed neural tube–like structures without cortical layering and containing ChAT^+^ neurons, consistent with spinal cord identity (Figure 9A).

We first assessed mature neuron populations in our SCO model of MS (Figure 9A). We performed an immunofluorescence for neuroblast marker DCX, glutamatergic neuron marker vGlut1 and GABAergic marker GAD67 (Figure 9A). Quantification revealed no significant change in neurogenesis in SCO from MS patients (Nested one-way ANOVA, *p* = 0.1862).

In contrast, a significantly higher number of GABAergic neurons was observed (Nested one-way ANOVA, *p* = 0.0074), especially in SPMS (*p* = 0.0066) compared to control, while no significant differences were detected for glutamatergic neurons (Nested one-way ANOVA, *p* = 0.3277) (Figure 9B). In c-organoids, we observed a significant increase in the excitatory marker VGLUT1 without changes in GAD67, suggesting a relative shift toward excitatory signaling. In contrast, in our SCO, GAD67 expression was significantly increased whereas vGLUT1 marker remained unchanged, indicating region-specific alterations in neuronal subtype marker expression rather than a uniform excitatory/inhibitory imbalance. An qualitative analysis of ChAT^+^ motor neurons did not highlight any difference in ChAT^+^ motoneurons expression in MS SCO compared to control (Figure 9A).

We also analyzed glial cell populations in SCO by immunofluorescence for astrocyte marker GFAP and oligodendrocyte marker Olig2 (Figure 9A). Quantifications revealed a significant difference in astrocytic cells population between groups (Nested one-way ANOVA, *p* = 0.0326). A significant decrease in astrocytic cells was detected in PPMS (*p* = 0.0180) compared to control. No significant difference was detected in Olig2^+^ cell population between groups (Nested one-way ANOVA, *p* = 0.5619) (Figure 9C).

To assess oligodendrocyte myelination capacity in our SCO model of MS, we performed immunofluorescence staining for Olig2 and MBP. The proportion of oligodendrocytes that matured into myelinating oligodendrocytes was determined by quantifying the ratio of Olig2^+^MBP^+^ cells relative to the total number of Olig2^+^ cells. Quantification revealed a significant overall difference among groups (Nested one-way ANOVA, *p* = 0.0416) with a significant decrease in myelinating oligodendrocyte population detected in PPMS (*p* = 0.0471) compared to control (Figure 9C).

Finally, we analyzed p21 expression (Figure 9D) and detected a significant overall difference among groups (Nested one-way ANOVA, *p* = 0.0122) with a decreased expression in PPMS (*p* = 0.0286) and a non-significant decrease in RRMS (*p* = 0.0570) SCO, similar to the decrease of p21 observed in our c-organoids.

## Discussion

We show that long-term cultured c-organoids and SCO from MS patients exhibit alterations in oligodendrocyte differentiation and maturation, with a marked reduction in myelination capacity specifically observed in PPMS organoids. In parallel, an imbalance of glutamatergic/GABAergic neurons associated with a decrease in the astrocytic population was observed. We linked these effects to the dysregulation of the CDKi p21 and its regulators E2F1 and PAK1. Our findings demonstrate that in MS, inherent deficits in the p21 pathway may alter glial and neuronal cell populations and may contribute to the pathogenesis and clinical manifestations of the disease.

In this study, we developed and used 20 distinct patient-derived iPS cell lines and 3 clones were generated from each iPS cell line to validate the reliability of our observations. iPSC cell lines were derived from 6 controls and 14 MS patients of different ages (from 23 to 67 years old), genders, and ethnicities, to ensure data reliability. Statistical analysis confirmed there were no differences in age or gender distribution between the groups (Tables 1, 2) and all iPS cell lines passed quality control and karyotype analysis.

Organoids generate oligodendrocytes and myelin after 20 to 30 weeks (Matsui et al., 2018) that respond to promyelinating drugs (Renner et al., 2017; Madhavan et al., 2018). Therefore, we decided to culture our organoids until day 120–150. At this time-point we observed mature neuronal populations such as GABAergic and glutamatergic neurons, mature glial cells such as astrocytes and myelinating oligodendrocytes (Figures 2C,E). Furthermore, we detected a strong colocalization of the myelin marker MBP and the neuronal marker DCX in control organoids, suggesting myelination of neurons (Figure 7). These long-term cultured organoids are a novel tool to study the genetic contribution on oligodendrocyte maturation and myelination capacity, and potentially to better understand the neurodevelopmental aspects of MS.

In PPMS, a progressive and irreversible decline in neurological function occurs despite the use of anti -immune/inflammatory treatment. It is proposed that inherent dysfunction in OPCs and/or oligodendrocytes may play a role in the pathogenesis of PPMS (Lassmann et al., 2012). In our c-organoid model, we observed a significant reduction in Olig2 expression, accompanied by a marked decrease in Olig2^+^ APC^+^ and Olig2^+^ O4^+^ MBP^+^ cells in PPMS. These findings indicate not only a reduction in the oligodendrocyte lineage, but also in mature myelinating oligodendrocytes. In our SCO model, while the decrease in Olig2 expression in PPMS was modest, we still detected a significant loss of mature myelinating oligodendrocytes. This suggests two distinct pathogenic mechanisms in PPMS. In the brain, we hypothesize that OPCs are reduced leading to a lower number of mature myelinating oligodendrocytes, whereas in the spinal cord, the total oligodendrocyte population is not dramatically affected, only the number of mature myelinating oligodendrocytes is reduced, indicating a defect in oligodendrocyte maturation.

PPMS iPSC-derived oligodendrocytes display widespread transcriptional alterations affecting cell adhesion, apoptosis, and inflammation (Plastini et al., 2022). Single-cell transcriptomic analyses show reduced oligodendrocyte numbers and increased expression of immune-related genes in oligodendrocyte lineage cells and astrocytes from PPMS patients (Clayton et al., 2024). Functionally, PPMS-derived neural progenitors also fail to confer neuroprotection in demyelination models (Nicaise et al., 2017), suggesting a convergence of molecular and functional impairments in PPMS.

In our previous work, we identified p21 as a crucial protein in the pathogenesis of progressive MS, given its role in oligodendrocyte differentiation and the hypomyelination observed in p21 knockdown mice (Zezula et al., 2001). Beyond its function in myelination, p21 acts as an autoimmunity suppressor, with p21^−/−^ mice developing a lethal autoimmune syndrome characterized by the production of pathogenic autoantibodies (Santiago-Raber et al., 2001; Santiuste et al., 2010). In this study, we observed a sustained decrease in p21 expression at both day 42 and day 120 in PPMS-derived organoids, consistent with previous findings and indicating that this dysregulation is not transient. Although p21 expression was reduced in all MS organoids at D42, this decrease persisted at D120 only in PPMS organoids, suggesting an early shared cell cycle dysregulation followed by subtype-specific divergence during long-term maturation. Together with our previous report of early defects in oligodendrocyte differentiation (Daviaud et al., 2023), these findings support the idea that sustained p21 impairment in PPMS may contribute to reduced oligodendrocyte maturation and myelination in both brain and spinal cord organoids, potentially underlying the remyelination failure characteristic of PPMS.

However, little is known about the implication of other CDK inhibitors on MS. Therefore, we analyzed the expressions of p16, p57, and p27, as they have documented roles in oligodendrocyte proliferation, differentiation capacity, and myelination (Casaccia-Bonnefil et al., 1997; Larocque et al., 2005; Kremer et al., 2009; Jablonska et al., 2012; Jadasz et al., 2012) and because they have been linked to several neurodegenerative disorders, including MS (Nicaise et al., 2019). In our work, we did not detect any difference in p16, p27 or p57 expression in MS organoids compared to control. Marker location, expression and cell morphology were nearly identical in every condition (Figure 3). Only p21 expression was significantly lower in MS organoids as previously described (Daviaud et al., 2023). However, when we performed the same analysis in iPSC derived NPCs, we detected a change in p16 expression in PPMS compared to control, similar to the result obtained by Nicaise’s team (Nicaise et al., 2019), while no difference was observed for p57, p27 or p21. This result seems to indicate that NPCs *in vitro* and NPCs growing in organoids have different regulations and behaviors that will need further characterization.

We also studied p21 regulators E2F1 and PAK1. E2F1 is required for the activation of the p21 gene through Ras (Gartel et al., 2000; Radhakrishnan et al., 2004), act as a stemness regulator in neural stem cells (Cooperkuhn et al., 2002) but also regulate OPCs differentiation into oligodendrocytes, thereby stimulating myelination (Magri et al., 2014). Moreover, a link between E2F1 and MS has been described. Natalizumab, a FDA-approved treatment for MS induces the downregulation of miR-17, leading to an upregulation of E2F1 and p21 target genes (Meira et al., 2014). We did not observe any difference in E2F1 expression at D42 but did see a significant decrease at D120 in MS organoids, particularly PPMS, compared to control (Figure 5). These results suggest that E2F1 may not be involved in stem cell dysregulation, or cell cycle exit, but may play a role in later stages, such as oligodendrocyte differentiation and maturation in our paradigm. p21-activated kinases (PAKs) are Cdc42/Rac–activated serine–threonine protein kinases that can positively regulate oligodendrocyte differentiation and are required for the correct formation of myelin sheaths in the CNS (Brown et al., 2021). Interestingly, PAK1 can block p21 expression (Prudnikova et al., 2016). We detected a significant increase of PAK1 expression at D42, particularly in PPMS and RRMS compared to control. The dysregulation of oligodendrocyte differentiation and maturation may result from a synergy between p21 and E2F1 dysregulation, as both proteins are essential for proper oligodendrocyte maturation and myelination, with E2F1 also controlling p21 expression. While an increase in PAK1 could promote oligodendrocyte differentiation, the PAK1-induced inhibition of p21 expression may counteract this effect by obstructing OPC differentiation into oligodendrocytes, further compounded by the reduction in E2F1 expression, which is likewise crucial for proper oligodendrocyte differentiation. Further experiments are needed to confirm this hypothesis.

Astrocytes play a vital role in the central nervous system (CNS) through their multifaceted functions. They secrete factors that support neuronal survival and synaptogenesis, while also influencing OPCs. Furthermore, astrocytes contribute to myelination, a process requiring intricate cellular cross-talk within the CNS (Kıray et al., 2016). We detected a significant decrease in astrocyte marker intensity in MS organoids, suggesting a decrease in glial cell differentiation (Figure 7). Interestingly this reduction of GFAP expression was detected in both our CO and SCO models of MS. This result suggests that an innate disruption of astrocyte proliferation/differentiation capacity may participate to MS pathogenesis. However, it has been suggested that reactive astrocytes change their morphology and size according to their reactive state in vitro and *in vivo* (Xiong et al., 2022), and thus the difference we detected in GFAP marker intensity could be due to a decrease in astrocyte size after activation in a pro-inflammatory mode. Although not specifically anticipated, the reduction in GFAP^+^ astrocytes in PPMS organoids was biologically plausible. Glial cells express ubiquitous PAK isoforms (Civiero and Greggio, 2018), and regulators upstream of p21 are known to influence neural stem cell maintenance and lineage specification (Gartel et al., 2000), suggesting that perturbations in this pathway may affect multiple glial populations. Consistent with this, single-cell transcriptomic studies in PPMS report alterations in both oligodendrocyte lineage cells and astrocytes (Clayton et al., 2024), supporting the concept of broader glial dysregulation. Further analysis of astrocyte properties in organoid model of MS will help to better understand their role in MS.

We also detected an imbalance in GABAergic/glutamatergic neurons. In our CO model, we did not observe a significant change in the GABAergic neuron population, but we did detect a significantly higher expression of glutamatergic neuron markers in PPMS and RRMS (Figure 7). Abnormal glutamate levels have been found in MS patients with increased levels in active lesions (Srinivasan et al., 2005), and glutamate excitotoxicity has been described as an important mechanism in autoimmune demyelination (Pitt et al., 2000) associated with worsening of MS symptoms, particularly related to cognitive functions (Azevedo et al., 2014). In our SCO model, we detected an increase in GABAergic neuron marker, while no important change in glutamatergic marker was detected. Interestingly a reduced concentration of GABA has been associated with physical disability and fatigue in patients with MS (Cawley et al., 2015; Cao et al., 2018; Arm et al., 2021) and modulation of GABA neurotransmission may be an important target for neuroprotection in multiple sclerosis (Cawley et al., 2015). Interestingly, astrocytes can regulate neurotransmitter homeostasis, as they uptake released neurotransmitters, such as glutamate and GABA, and the balance between excitatory and inhibitory influences protect neurons from excitotoxic cell death (Mahmoud et al., 2019). In our organoid model, we detected a decrease in astrocyte population associated with an imbalance of GABAergic/glutamatergic neuron population. These results may suggest an important underlying mechanism for the clinical manifestations of cognitive impairment, physical disability or fatigue described in patients with MS. Organoids may thus be an experimental model to study potential GABAergic/glutamatergic agonists and modulators as potential treatments for MS.

We also used our SCO model of MS to study spinal cord motor neurons as motor neurons may be reduced in postmortem MS patients (Vogt et al., 2009) and also in EAE rodent model (Gilmore et al., 2009) are implicated in MS pathology. However, we did not detect any change in ChAT^+^ motor neurons in our SCO, suggesting that the motor neuron degeneration if present in MS may be secondary to inflammation.

Although organoids provide valuable insights into the study of MS, challenges remain. A known challenge in organoid-based models is inter-line and intra-line variability. In our study, organoids were derived from 20 independent donor cell lines, with multiple clones and independent differentiation batches generated per line. All lines efficiently reprogrammed into stable iPSCs and consistently formed c-organoids and SCO without overt differences in growth, size, or gross morphology. While some degree of variability between organoids and batches was observed, as expected in 3D culture systems, this variability was comparable across control and MS groups and did not obscure the disease-associated phenotypes. Importantly, the observed alterations were reproducible across independent donor lines within each subtype, suggesting that the reported effects are not driven by a single line. Although variability remains an inherent feature of organoid systems, recent protocol refinements have substantially improved reproducibility (Sivitilli et al., 2020), with variability approaching that observed in human brain tissue.

Organoids generated using our protocol lack microglia, which are crucial components in MS research. Interestingly some such models have been developed and showed improved neurogenesis and neuroprotection (Park et al., 2023; Chen et al., 2025). The absence of vascularization represent another limitation as it impairs nutrients and oxygen delivery to cells within the organoids, resulting in cellular stress, oxygen deprivation and necrosis (Kim and Chang, 2023). Further experiments are needed to implement mature microglia and vascularization in organoids to provide a better human cellular model for studying neuron–glia and glia–glia interactions in brain development and the pathogenesis of MS.

Another limitation is that organoids recapitulate early stages of human neurodevelopment, whereas MS typically manifests between 20 and 40 years of age. As iPSCs undergo epigenetic rejuvenation during reprogramming, organoid models reflect embryonic-like developmental stages rather than the biological age of patients at disease onset. However, this feature makes organoids particularly suitable for investigating intrinsic and developmentally rooted susceptibilities that may contribute to MS pathogenesis. In this context, our model allows the exploration of early alterations in neural and oligodendroglial lineage specification that could predispose individuals to later disease manifestation. Notably, the existence of pediatric-onset MS, with approximately 5,000 affected children and adolescents in the United States and 30,000 worldwide (Yeh et al., 2009), further supports the relevance of examining early neurodevelopmental vulnerabilities and genetic contributions to MS.

Organoid models are proving to be highly valuable tools across various domains of research. They may be valuable in evaluating the neurotoxicity of drugs and studying the role of environmental factors, such as EBV infection. By enabling personalized drug testing on patient-derived models, they introduce the innovative concept of “clinical trials in a dish” (Smirnova and Hartung, 2024). Although still in early stages, organoid research holds immense potential for tailoring treatments and improving therapeutic outcomes.

In the context of multiple sclerosis (MS), cerebral and SCO offer unique advantages. At early stages, they are useful for studying precursor cell proliferation and differentiation, while at later stages, they provide insights into neural and glial cell maturation, as well as myelination. Our findings highlight the involvement of the p21 pathway in MS, along with its regulators E2F1 and PAK1. Dysregulation of these proteins leads to reduced oligodendrocyte differentiation, maturation, and myelination capacity. It also disrupts astrocyte populations and causes an imbalance in inhibitory and excitatory neurons—key factors in MS onset and symptom progression. These novel organoid models will play a pivotal role in unraveling the complex interplay between genetic susceptibility and environmental factors in MS, offering a comprehensive approach to understanding its multifaceted nature.

## Data Availability

The original contributions presented in the study are included in the article/supplementary material, further inquiries can be directed to the corresponding authors.
